# Monetary Equivalent Value (MEV) of a Published Article in Psychology

**DOI:** 10.5964/ejop.v15i2.1595

**Published:** 2019-06-07

**Authors:** Joseph G. Ponterotto

**Affiliations:** a Division of Psychological & Educational Services, Graduate School of Education, Fordham University – Lincoln Center, New York, NY, USA; Webster University Geneva, Geneva, Switzerland; Maria Grzegorzewska University, Warsaw, Poland

**Keywords:** monetary equivalent value (MEV), impact factor, research, psychologists’ salaries, psychologists’ work hours

## Abstract

Publishing one’s research in peer-reviewed journals is generally acknowledged to be a valuable enterprise. This is particularly the case for academic and research psychologists who rely on publications for career status, stability, and advancement. Psychological researchers can devote extensive amounts of time to planning, conducting, writing up, and getting their research published in respected psychology journals, yet their work efforts in this regard have heretofore never been quantified monetarily. This article introduces the concept of a monetary equivalent value (MEV) of a published article in psychology. An initial basic linear equation is introduced that sets the dollar (or Euro) value of an article based on the median number of hours involved in publishing an article, the mean hourly wage of psychologists, and the 5-year Impact Factor (IF) of the journal in which the article is published. MEVs were calculated for the full range of journals published by the American Psychological Association (APA) that have IF ratings. MEV values varied widely, from a low of $4,562 for an article published in the journal "Dreaming", to a high of $131,613 for an article appearing in "Psychological Bulletin". This article represents the first to explore the MEV as an additional metric to understand the impact of published articles, and as such this exploratory study has numerous limitations. Chief among these is the study’s reliance on the controversial Journal Citation Reports (JCR) journal impact factor metric, as well as its extrapolation from a limited medical literature on the average number of hours involved in publishing a study.

The importance and value of publishing research in peer-reviewed journals in psychology is widely acknowledged in the profession. For academic psychologists in tenure-tract college and university positions, and for psychologists employed in other research-weighted settings such as major hospitals, research institutes, and community mental health centers, publishing one’s work is often essential in securing career stability and mobility, status among colleagues, and career advancement (see Pfleegor, Katz, & Bowers, 2017). Publishing one’s research in “known” and respected journals marks a “success point” and a wider professional acknowledgement of the importance of one’s investigative efforts. Furthermore, from the institutional perspective, publications in respected academic journals bring international prestige and promotes the recruitment of new promising staff and students (see Doyle & Cuthill, 2015).

Psychological researchers spend significant amounts of time planning, conducting, writing-up, and submitting their research studies. Components of the process include conceiving an idea and conceptualizing the project; selecting collaborators and co-authors and delineating team member responsibilities; conducting and integrating the literature review; setting the research paradigm and statistical models; locating and securing research measures and protocols; preparing and securing Institutional Review Board (IRB) approval for the study; data collection; data cleaning and data analysis; manuscript preparation; selecting an appropriate journal and preparing for submission; responding to Reviewer comments and revising the manuscript; preparing the revision cover letter; and copy-editing and final page proof review. In total, researchers can spend hundreds of hours to see a single study through to publication in a peer-reviewed psychology journal (Song, Abedi, Macadam, & Arneya, 2013). What material value should be placed on this effort? More specifically, what is the equivalent monetary value of all the work that goes into getting a research study published in a peer-reviewed journal?

This article introduces the exploratory concept of the Monetary Equivalent Value (MEV) of a published article in a peer-reviewed psychology journal. A basic linear equation is presented that establishes a dollar (or Euro) value of a published article in psychology based on 1) the median number of hours it takes to publish an article from conceptualization to final publication, 2) the mean hourly wage of psychologists, and 3) the 5-year Impact Factor (IF) of the particular journal in which the article is published. It should be noted that there is no research assessing the average number of hours it takes to publish different types of journal articles in psychology, and the present MEV model borrows from a limited medical research literature. Further it should be noted at the outset, that the cultural and economic context for the present study is primarily anchored within the North American sphere, and international replication, adaptation, and expansion of methods described herein is highly encouraged.

As the MEV formula relies heavily on Impact Factors set by the Institute for Scientific Information’s (ISI) Journal Citation Reports (JCR), published by Clarivate Analytics (formerly Thomson Reuters), it is important to begin the discussion with a review of the benefits and limitations of the bibliometric IF.

## Value and Limits of the Impact Factor

There is something alluring about rankings and ratings, a standard metric for easy comparisons. Take, for example, the popularity of the annual *U.S. News & World Report*’s “Best Colleges Ranking.” College Deans and other university administrators often keep close track of the rankings with the goal of either maintaining or raising their ranking in their respective university or college categories. Top ranked universities often highlight their ranking in advertisements or profiles of their university in both social and print media.

In the area of professional journal prestige, perhaps the most popular and often cited rating or bibliometric is the journal’s Impact Factor (IF) (Chorus & Waltman, 2016). Anchoring the IF is the proportion of citations to articles in the journal relative to the number of articles published in the journal over specified time frames. Journal editors across disciplines, particularly in the physical, behavioral, and social sciences, often call attention to the importance of IFs. Take, for example, the following quotes from two journal editorial statements. The first is from Cuellar (2016), opening a special issue on IFs for the *Journal of Transcultural Nursing*, where the editor noted the value of IFs. The second is written by Reich-Erkelenz, Schmitt, and Falkai (2016) and expresses pride in the latest IF rating for their journal, *European Archives of Psychiatry and Clinical Neuroscience*,

“Publishing in a journal with an impact factor means that scholars are reading the journal and citing it in their work. It is the number of times that an article is being cited in someone else’s articles. It means that the articles that we are publishing uphold scientific integrity and are being used to help other scientists to advance their work. It means that our work is getting out to others by not just having someone read them but by saying ‘go read this’ as a citation in someone else’s work.” (Cuellar, 2016, p. 437)

“We are proud of opening this issue with awesome news: ISI has just released the new impact factor list for 2015, according to which European Archives of Psychiatry and Clinical Neuroscience (EAPCN) for the fourth time in a row has risen its impact factor and now has achieved a rank of 4.113, thus for the first time negotiating the hurdle of 4.0.” (Reich-Erkelenz et al., 2016, p. 475)

Such quotes by editors are not uncommon (see also Cacchione, 2017), and various journals state their IFs on the journal’s homepage. IFs have taken on a level importance and status that may or may not be warranted depending on the use of the bibliometric. For psychologists and scientists in more developing countries, publishing in higher IF journals promotes aspirations of joining the larger scientific community (see Mishra & Neupane, 2018).

The rationale and use of IFs have evolved over the last 90 years. Gross and Gross (1927) first suggested that the reference count could be used to rank the use of scientific journals. The term “impact” was introduced by Garfield (1955), who is often credited with the concept of “impact factor,” though “factor” was not added until the 1961 Science Citation Index (Garfield, 1963, 1996). The initial intent of the IF was to help librarians compare the quality of diverse journals within particular scientific disciplines (Garfield, 1955; Kiesslich, Weineck, & Koelblinger, 2016). Such data could help in librarian decision-making in terms of which journals to subscribe to given available budgets. The journal IF was never intended to evaluate the merits of a particular article or the scholar(s) publishing the article. However, in recent decades, the use and interpretation of journal IFs have expanded markedly beyond their original intent, even to the point of impacting researchers’ neural reward signals. A fairly recent fMRI study of 35 neuroscientists from the fields of psychology, psychiatry, and neurology, showed significantly greater neural activity in the nucleus accumbens (NAcc; the reward signal center of the Cerebrum) of these scientists when responding to a stimulus of potentially publishing in a high IF neuroscience journal over a medium or low IF journal (Paulus, Rademacher, Schafer, Muller-Pinzler, & Krach, 2015). This laboratory study provided the first evidence that consideration of journal IFs can effect neural response patterns, and further demonstrates “how deeply entrenched the concept of [journal] IF has become on the neural systems level” (Paulus et al., 2015, p. 11).

Are IFs worthy of the attention and significance they now garner across the sciences? Concern for the inappropriate interpretation of IFs in various contexts, for example, evaluating the merits of an individual article, or researchers themselves, or the collective faculty within a university department, or merits of grant applications, has led to a broad body of research highlighting the limitations and potential manipulations of IFs. A concise summary of these limitations is provided in the San Francisco Declaration on Research Assessment (DORA; 2012), which emanated from a working group of editors and publishers of scholarly journals that met during the 2012 annual meeting of the American Society for Cell Biology in San Francisco. The overarching recommendation of the working group of scholars which was endorsed by 74 renowned scientists worldwide was as follows:

“Do not use journal-based metrics, such as Journal Impact Factors, as a surrogate measure of the quality of individual research articles, to assess an individual scientist’s contributions, or in hiring, promotion, or funding decisions.” (DORA, 2012, p. 2)

The San Francisco Declaration on Research Assessment (DORA, 2012) and a host of independent researchers have highlighted some of the limitations of using IFs for the purpose of assessing and comparing the scientific merit or output of institutions or individual researchers. Collectively, these limitations can be organized along six content areas. First, citation distributions within journals are highly skewed (DORA, 2012; Olff, 2014), and the IF is not representative of the number of citations that individual articles accumulate within the same journal (deLeon, 2018; Eyre-Walker & Stoletzki, 2013). Second, the properties of the journal IF are field specific, and citation sources can vary widely in article type such as empirical research, substantive review, or theoretical paper (DORA, 2012). Furthermore, IF magnitudes vary widely across disciplines, making cross-disciplinary comparisons of scientific merit or impact virtually impossible (Liu, Gai, Zhang, & Wang, 2015; Postma, 2007). For example, the highest IF for a psychology journal in this study is for *Psychological Bulletin*, with the latest-year IF rating of 16.793; whereas the highest rated medical journal, *The New England Journal of Medicine*, reached an IF of 72.406 in its last year (National Institute of Environmental Health Sciences, 2018).

A third limitation of journal IFs is that they can be manipulated (or “gamed”) by editorial policy (DORA, 2012). More specifically in this regard, journals editors (and review boards) can raise the impact factor by accepting fewer articles for publication in the journal, thus increasing the citation to publication ratio (Kiesslich et al., 2016). Fourth, the journal IFs do not exclude self-citations, and by promoting self-citations, journals, or authors writing for specific journals, can boost IFs (Chorus & Waltman, 2016; Liu et al., 2015). Fifth, online-to-print delays in article publication can artificially raise IFs, particularly for longer print delays as IFs are mainly based on date of publication in print form (Tort, Targino, & Amaral, 2012). Sixth, according to DORA: “data used to calculate the Journal Impact Factors are neither transparent nor openly available to the public” (DORA, 2012, p. 1). Access to Clarivate’s Journal Citation Reports (JCR) and their IF ratings, or Scopus’s SCImago journal ratings require expensive subscriptions only available to researchers or the public with institutional access to these sources.

Despite the known limitations of relying on IFs as measures of individual or institutional merit, they hold some value in assessing impact. Eyre-Walker and Stoletzki (2013) examined three methods of assessing the merit of an empirical paper: independent scholar rating of the paper post publication; the number of citations the paper accrued within 6 years after publication; and the IF of the journal in which the study appeared. Though there was a moderate correlation (*r* = .38; medium effect size) between scholar ratings of the published article and the number of citations eventually accrued by the article, the scholars over-rated (i.e., were influenced by) papers in high IF journals. When this favorability bias was controlled for statistically, the correlation was negligible (*r* = .15, small effect size). Eyre-Walker and Stoltezki (2013) suggested that “scientists have little ability to judge either the intrinsic merit of a paper or its likely impact” (p. 1). The authors concluded that while none of the three methods is a good measure of merit, the IF may be the most satisfactory given it is a pre-publication measure.

Acknowledging that IFs do not describe the merits of an individual article, but instead acknowledge the impact and influence of the journal itself on its target discipline, this bibliometric serves a useful limited purpose (see also Friedman, 2016; Postma, 2007). With the proliferation of print and online professional journals, IFs do provide some standard metric on the article citation to publication ratio. Most researchers would prefer their articles be published in journals that are often cited by other scholars within their own fields. Of course, at times weaker articles do reach publication in high impact journals, and very good articles appear in lesser known and cited journals.

As highlighted by Garfield (1996), “In the final analysis, impact simply reflects the ability of journals and editors to attract the best papers available” within their discipline (p. 411). Certainly, researchers would like to see (at least some of) their research and major conceptual/review/theoretical articles published in the journals that attract the best papers. The reality is that despite the known limitations of IFs, articles published in high IF journals have greater value in that they are more often cited by other researchers. It is likely then, that researchers who have a good number of published articles in high impact journals, will garner a collectively greater professional status.

The primary purpose of this study is not to further debate the merits and limits of bibliometrics, particularly in our case, the Impact Factor (IF). The purpose is to stimulate thought and reflection on the material monetary value-equivalent to the extensive amounts of work researchers devote to the research and publication process. IFs can be quite abstract to members both within and outside the scientific community, whereas the concept of money, or monetary value, may be more easily understood by those attempting to understand the value and work involved in publishing articles in select journals. A second purpose of this initial exploratory study is to stimulate follow-up research, from different national, statistical, and conceptual angles, on the Monetary Equivalent Value (MEV) formula as an additional metric to consider when weighing the impact of a published article in psychology.

## Method

### Median Number of Hours Involved in Publishing a Research Article

A review of the psychology literature did not uncover any research calculating the average number of hours involved in publishing a study. However, in the field of medicine, such a study was conducted. Song et al. (2013) had 13 surgeons specify time on task for 171 studies that they had published from 1990 through 2012. The studies focused upon involved retrospective designs rather than randomized control trials, given the much greater frequency of retrospective studies (Song et al., 2013). The number of hours per study ranged from 29 hours to 1,287 hours. Given the positive skew of the distribution the researchers calculated the median number of hours per study which was 177 hours. This total represents the efforts of the collective team, not just the lead author. Naturally, the distribution of work tasks and hours across co-authors or team members will vary dependent on the size of the research team and scope of the study conducted. Table 1 summarizes the results of the Song et al. (2013) study organized along the median number of hours involved in eight components of the research and publication process. The greatest amount of time was devoted to data collection (22% of total time), followed by manuscript preparation (22%).

**Table 1:** Median Number of Task Hours Spent on a Research Article from Idea Conceptualization to Publication Based on Song et al.'s (2013) Study

Component of Study	Median Hours	Percent of Total
Pre-Study Planning	20	11
Literature Review	21	12
Ethics Process	7	4
Data Collection	41	23
Data Analysis	22	12
Manuscript Preparation	39	22
Manuscript Submission	10	6
Manuscript Post-Submission	17	10
Total	177	100

Though the Song et al. (2013) study was in medical surgery research, and not in psychology, aspects of the positivist and post-positivist research paradigm, the scientific method, and the associated quantitative procedures transcend both medical and psychological research (see Ponterotto, 2005). Furthermore, as in medical research, retrospective studies are much more common than randomized control trials in many subfields of psychology research (Heppner, Kivlighan, & Wampold, 2007). Thus for the purpose of this study, and until the Song et al. (2013) study is replicated in various subfields of psychology, we will use the median hours per study of 177 in the present calculations. However, it should be emphasized that until researchers replicate the Song et al. (2013) study across various psychology journal contexts, the current MEV discussion is a conceptual proposal and should not be implemented in any journal evaluation procedure.

### Mean Hourly Wage of Psychologists

As with the number of hours involved in publishing a study, the salary of psychologists can vary widely. According to the U.S. Government’s *Occupational Outlook Handbook* (Bureau of Labor Statistics, U.S. Department of Labor, 2018), the median pay for psychologist in 2016 (latest data available) was $75,230, or $36.17 per hour based on a 40 hour-work week and 52 weeks per year. The mean salary for academic psychologists in the 2016–2017, according to the American Psychological Association (Christidis, Lin, & Stamm, 2018) was $74,085, or $35.62 per hour (40 hour-work week). For the present study we used the mean of the two salary reports, which fell at $35.9, rounded to $36. Therefore, for the purposes of the present study, the average hourly wage for psychologists is set at $36.

### Determined Value of a Published Article in Psychology Independent of Journal IF

To calculate the monetary value of modal article in a psychology journal, we used the equation: (Median number of hours for study) (average hourly wage of psychologists), or in US dollars, (177) ($36) = $6,372. The equivalent value in Euros (as of the January 20, 2018 exchange rate) where $1 USD = 0.8184 €; $36 X 0.8184 = € 29.46; and (177) (€ 29.46) = € 5,214 article monetary value.

### Impact Factors and Journal Selection

Naturally, the hypothetical dollar value of a published study will be impacted by the quality or reputation of the journal in which the study is published. The popular, though controversial bibliometric of the 5-year Impact Factor (IF) established by the Institute for Scientific Information (ISI) and published in the annual Journal Citation Reports (Clarivate Analytics, formerly Thomson Reuters) was selected for the present study.

A journal’s Impact Factor (IF) in a given year is the number of citations to the journal in that year to articles published in the journal during the previous 2 years (numerator) divided by the number of citable articles (substantive articles and reviews only) published in the journal in the preceding 2 years (denominator) (Garfield, 2006). The 5-year IF bibliometric extends this formula back 5 years, rather than two.

For an initial sample of journals, the author selected all journals affiliated with the American Psychological Association (APA) that reported IF ratings (some of the newest journals had not yet reported IFs). If available, we utilized the Journal Citation Report (JCR) 5-year Impact Factor (IF) ratings (in two cases where the 5-year IF was not available, we incorporated the latest IF rating). On APA’s webpage (www.apa.org), under the *Browse Journal* link, the IFs are listed with the journal descriptions.

### Final MEV Linear Equation

The final linear product equation in calculating the monetary equivalent value (MEV) of a published article in psychology was:

MEV = (M) (W) (IF)

Where M = the medium number of hours involved in publishing a research article (or 177 in the present study); where W = the average hourly salary of psychologists (or $36 for present purposes); and where IF = the 5-year impact factor of the selected psychology journal. The equation is operationalized across the variety of journals comprising Table 2. The present author would like to again emphasize that the JCR IF is controversial in the scientific literature (e.g., DORA, 2012), and the average number of hours involved in publishing a study, as well as the average hourly salary of psychology researchers is based on a limited literature and initial estimates.

**Table 2:** Journals of the American Psychological Association, Classification Category, Rank Within Category, 1- and 5-Year Impact Factors, With Equivalent Dollar and Euro Value as of 2018 Ranked by Monetary Value^a^

Journal	Category	Rank	1-Year IF	5-Year IF	$ Equi	**€** Equi
Psychological Bulletin	Psych. – Multidis.	2 of 128	16.793	20.655	131,613	107,712
Psychological Methods	Psych. – Multidis.	10 of 128	4.667	10.141	64,618	52,883
Psychological Review	Psych. – Multidis.	5 of 128	7.638	9.506	60,572	49,572
American Psychologist	Psych. – Multidisc.	7 of 128	6.681	7.329	46,700	38,219
Jour. of Pers. & Soc. Psych.	Psych. – Social	4 of 62	5.017	7.296	46,490	38,047
Jour. of Applied Psychology	Psych. – Applied	5 of 80	4.130	6.890	43,903	35,930
Jour. of Consult. & Clin. Psych.	Psych. – Clinical	8 of 121	4.593	6.053	38,570	31,366
Jour. of Exp. Psych.: General	Psych. – Exper.	4 of 84	4.420	5.883	37,487	30,679
Jour. of Abnormal Psychology	Psych. – Clinical	11 of 121	4.133	5.722	36,461	13,472
Jour. of Educational Psych.	Psych. – Edu.	5 of 58	3.459	5.240	33,389	27,326
Developmental Psychology	Psych. – Develop.	17 of 70	3.228	4.653	29,649	24,265
Jour. of Occup. Hlth. Psych.	Psych. – Applied	16 of 80	2.679	4.534	28,891	23,644
Health Psychology	Psych. – Clinical	15 of 121	3.458	4.352	27,731	22,695
Emotion	Psych. – Exper.	12 of 84	3.251	4.266	27,183	22,247
Jour. of Counseling Psychology	Psych. – Applied	19 of 80	2.516	4.094	26,087	21,350
Psychological Assessment	Psych. – Clinical	18 of 121	3.307	3.922	24,990	20,452
Psychology and Aging	Gerontology	7 of 32	2.812	3.843	24,488	20,041
Neuropsychology	Psych. – Clinical	19 of 121	3.286	3.734	23,793	19,472
School Psych. Quarterly	Psych. – Educat.	6 of 58	3.256	3.317	21,136	17,298
Psych. of Addict. Behaviors	Substance Abuse	11 of 34	2.543	3.308	21,079	17,251
Pers. Dis.: Theory, Res., & Trtm.	Psych. – Clinical	31 of 121	2.606	3.249	20,703	16,943
Jour. of Exp. Psych.: Applied	Psych. – Applied	26 of 80	2.156	3.224	20,543	16,812
Psychotherapy	Psych. – Clinical	32 of 121	2.573	3.216	20,492	16,771
Psychology of Violence	Psych. – Clinical	46 of 121	2.192	3.070	19,562	16,010
Jour. of Exp.Psych.: Lrn., Mem., & Cogn.	Psych. – Exper.	26 of 84	2.667	2.962	18,874	15,446
Law & Human Behavior	Law	7 of 147	2.822	2.919	18,600	15,222
Jour. of Exp. Psych.: Hum. Percp. & Perf.	Psych. – Exper.	32 of 84	2.287	2.833	18,052	14,774
Behavioral Neuroscience	Behav. Sciences	23 of 51	2.453	2.797	17,722	14,504
Psych of Aesth., Creat., & the Arts	Psych. – Exper.	46 of 84	1.860	2.751	17,529	14,346
Psych of Rel. & Spirituality	Psych. – Multidis.	40 of 128	2.000	2.732	17,408	14,247
Journal of Family Psychology	Family Studies	15 of 43	1.742	2.669	17,007	13,919
Cult. Div. & Ethnic Minority Psych.	Ethnic Studies	1 of 15	2.040	2.620	16,695	13,663
Exp & Clin. Psychopharm.	Psych. – Clinical	47 of 121	2.186	2.577	16,577	13,567
Review of General Psychology	Psych. – Clinical	54 of 121	1.492	2.450	15,611	12,776
Psych. Trauma: Thr., Res., Prct., & Pol.	Psych. – Clinical	68 of 121	1.584	2.405	15,325	12,542
Psych of Men & Masculinity	Psych. – Social	22 of 62	1.957	2.387	15,210	12,448
Jour. of Comparative Psychology	Psych. – Multidis.	32 of 128	2.268	2.342	14,923	14,923
Psych., Publ., Policy, & Law	Law	22 of 147	1.915	2.244	14,299	11,702
Rehabilitation Psychology	Rehabilitation	21 of 70	1.615	2.309	14,713	12,041
Amer. Jour. of Orthopsychiatry	Social Work	7 of 42	1.692	2.220	14,146	11,577
Intern. Jour. of Stress Manag.	Psych. – Applied	42 of 80	1.632	2.077	13,235	10,832
Psychological Services	Psych. – Clinical	71 of 121	1.544	2.073	13,209	10,810
Prof. Psych.: Res & Practice	Psych. – Multidis.	67 of 128	1.209	1.987	12,661	10,362
Sports, Exercise, & Perf Psych.	Psych. – Applied	31 of 80	1.930	N/A	12,298	10,065
Jour. of Exp. Psych.: Anim. Lrn. & Cog.	Psych. – Biological	8 of 14	2.132	1.866	11,890	9,731
Asian Amer. Jour. of Psych	Ethnic Studies	3 of 15	1.528	1.771	11,285	9,236
Canadian Psychology	Psych. – Multidisc.	57 of 128	1.429	1.767	11,259	9,214
Canadian Jour. of Exp. Psych.	Psych. – Exper.	74 of 84	1.055	1.741	11,094	9,079
Psychiatric Rehab. Jour.	Rehabilitation	42 of 70	1.037	1.532	9,762	7,989
Families, Systems, & Health	Pub, Env, Occ Hlth	107 of 157	1.219	1.408	8,972	7,343
Psychoanalytic Psychology	Psych. – Psychoan.	1 of 31	1.068	1.380	8,793	7,196
Group Dyn.: Theory, Res. & Prac.	Psych. – Social	47 of 62	1.073	1.358	8,653	7,082
Jour. of Diversity in Higher Ed.	Ed. & Educ. Res.	128 of 235	1.029	1.200	7,646	6,257
Jour. of Neurosc., Psych., & Economics	Psych. – Multidis.	68 of 128	1.188	N/A	7,570	6,195
Military Psychology	Psych. – Multidis.	91 of 128	0.734	1.174	7,481	6,122
Training & Education in Prof. Psych.	Psych. – Educat.	50 of 58	0.810	0.983	6,237	5,104
Canadian Jour. of Behav, Sciences	Psych. – Multidis.	114 of 128	0.404	0.883	5,626	4,604
History of Psychology	Hist. of Soc. Sci.	4 of 35	1.000	0.839	5,346	4,375
Dreaming	Psych. – Multidisc.	83 of 128	0.867	0.716	4,562	3,734

*Note.* IF = impact factor.

^a^Euro exchange values were calculated on January 20, 2018, when $1 = 0.8184 Euros. Exchange calculator found at https://www.investingt.com/currencies/eur-usd-converter

## Results

Table 2 presents a rank ordering of the dollar (and Euro exchange equivalent on January 20, 2018) value of an article published in 59 journals sponsored by the APA. All but two of these journals (*Journal of Neuroscience, Psychology, and Economics*, and *Sports, Exercise, and Performance Psychology*) reported 5-year IFs. The monetary value of articles published in the chosen journals varied markedly, from a high of $131,613 for a *Psychological Bulletin* publication to a low of $4,562 for an article published in the journal, *Dreaming* (both journals are classified in the category of Psychology—Multidisciplinary). The median monetary value across all journals was $17,408 (*M* = $22,311; *SD* = $19,381). The median 5-year IF was 2.751 (*M* = 3.57, *SD* = 3.07). For the 1-year IF, the median value was 2.156 (*M* = 2.66, *SD* = 2.34). The IFs rose from the 1-year to 5-year period for all journals save for two: *Dreaming* and the *History of Psychology*.

Additional journals with high monetary values in the Psychology—Multidisciplinary category were *Psychological Methods* ($64,618), *Psychological Review* ($60,572), and the *American Psychologist* ($46,700). High value journals in the category of Psychology—Applied were the *Journal of Applied Psychology* (individual article valued at $43,903) and the *Journal of Occupational Health Psychology* ($28,891). In the Psychology—Social category, the *Journal of Personality and Social Psychology* ($46,490) had the greatest monetary value, followed by *Psychology of Men and Masculinity* ($15,210). In the Psychology—Experimental category, the *Journal of Experimental Psychology: General* ($37,487) was the highest; in Psychology—Developmental, *Developmental Psychology* ($29,694) was highly valued; in Psychology—Clinical, the *Journal of Consulting and Clinical Psychology* ($38,570), ranked first; and in the Ethnic Studies category the journal *Cultural Diversity and Ethnic Minority Psychology* ($16,695) was the top ranked.

## Discussion

Psychological researchers usually devote a good amount of time to planning, conducting, writing-up, and publishing research studies. It is generally understood that publishing one’s research in peer-reviewed journals is a necessary component of career stability and success in many research-oriented settings internationally. However, little medical research, and no psychological research, exists that documents the actual number of hours spent in the production and publishing of various types of research articles, and no research has heretofore specified a material or monetary-equivalent value of such work and dedication. The proposed “Monetary Equivalent Value” (MEV) linear equation, while primarily exploratory at present, opens the discussion on the topic. As noted by an anonymous reviewer of an earlier version of this manuscript, “Money is less abstract [than IFs] and easier to imagine.”

The present study is limited by the conceptual foundation of the MEV which relies on central tendency measures of the number of hours worked by psychologists and their average income/hourly wages. Further, this study is limited to the North American research publishing context, and may or may not be generalizable to other continents and countries. Additionally, the MEV is also anchored in the popular JCR journal Impact Factor rating, which as has been noted, is open to debate and controversy. Consistent with the recommendation from DORA (2012), the MEV value is theoretical and applies to an average article in the specific journal, and not to any particular article.

The concept of MEV is an interesting one to ponder as past literature has not examined the monetary equivalents of the time and effort devoted to the publishing process in psychology journals. An important goal of this article is to stimulate follow-up research internationally on potential formulas, applications, and limitations of MEVs. To that end, the following research directions are presented to address the limitations of the present study and stimulate thinking on additional journal metrics.

First, research is needed to study the average number of hours involved in publishing psychological research. Furthermore, research is needed to examine the average amount of hours devoted to publishing different types of psychological research. For example, what is the variation in work hours involved in reaching publication in correlational studies, versus quasi-experimental studies, versus fully randomized controlled experiments? For that matter, what is the estimated hourly work involved in preparing and publishing a meta-analysis or conceptual/theoretical article? Also, how do time frames for publishing an article in a specialty journal (e.g., *Dreaming)* vary from those published in broad appeal journals (e.g., *Psychological Bulletin)*? Further, research can examine whether preparation and publication time of a study varies depending on the experience levels of the researcher. Also, with newer social internet tools to facilitate and expedite sampling (particularly in survey research) such as Mechanical Turk, Qualtrics, and Survey Monkey, how will sampling time frames be affected (refer back to Table 1)?

Second, this study calculated the average hourly wage of psychologists based only in North America. Salaries and forms of compensation for published research can vary widely within and across nations and across first- and third-world economies. Also, the pressure to publish in higher impact journals can vary from country to country and from institution to institution within countries. This topic needs to be studied from a wider context and it would behoove psychologists to partner with sociologists and economists in interdisciplinary research.

Furthermore, research can attend more specifically to the salary or work hour variance among psychologists within research communities. For instance, the average U.S. salary of an assistant professor in psychology across all institution types is $61,500 (in 2016–2017 survey) (Christidis et al., 2018) or $29.57 per hour for a 40-hour work-week. The average psychologist in private practice, however, who may charge $150 per hour, may earn $312,000 a year for their 40-hour work weeks. The loss in income for a private practitioner to take time away from their patients to conduct and publish a study is far greater than for an academic who often has a flexible work week and where research is part of the job description and allotted time within the work week. Naturally, many private practitioners or practitioners in diverse settings do publish in psychology journals.

Third, MEVs will need to be recalculated each year or every few years as updated information on psychologists’ salaries, work hours involved in publishing a manuscript, and the IF becomes available. For the many new psychology journals appearing each year, 5-year impact ratings are not yet available, thus researchers may want to rely on single or 2-year IFs in some calculations. Naturally, journals without current IF ratings should not be assessed with the MEV model. It should also be noted that while some online-only psychology journals, such as *PLoSONE Psychology* and *Frontiers in Psychology* do report IFs, many newer online journals have not yet joined the JCR system.

Fourth, it is suggested that follow-up research examine variations on the MEV and the variables used to calculate the coefficient. For example, more complex extensions of the MEV formula can incorporate page lengths of published articles (on the assumption, for example, that a 12 page article published in the *Journal of Consulting and Clinical Psychology* is more “valuable” than a three page article).

It is acknowledged that the present study is just a first step to introduce a new concept to the growing discourse on the prestige and status of publication outlets in psychology. Money is universally understood, and publishing does impact the financial status of researchers and their home institutions. It is hoped that the present study will stimulate follow-up research internationally.

### Concluding Comments

At times the general public may not understand the amount of work and effort devoted to research and publishing. To some, an academic psychologist teaches a few courses a semester, has summers off, takes frequent sabbaticals, and if tenured, has a relatively secure job for life. Though of course this perception is a marked simplification and stereotype, quantifying and placing a monetary value on our research efforts may inform the public of researchers’ commitment to their profession and society. Using a concept such as monetary value in addition to traditional impact factor ratings to understand the status of journals and the studies they publish, may demystify the process for the lay public and increase the public’s accessibility to the psychology profession. Naturally, as an initial explication of a formulaic system (MEV) to consider a monetary metric, systematic follow-up research is called for before such a metric can be used with good levels of confidence. Many of the IF critics cited in this article would agree that it behooves the advancement of scientific fields to have multiple metrics to judge an article’s impact, and that an overreliance of a single metric, such as IF, should be avoided. It is the author’s hope that this article stimulates research on alternative evaluative metrics of journals in psychology.
